# Material Evidence of Sediments Recovered from Ancient Amphorae Found at the Potaissa Roman Fortress

**DOI:** 10.3390/ma16072628

**Published:** 2023-03-26

**Authors:** Iulia Alexandra Farcas, Thomas Dippong, Ioan Petean, Marioara Moldovan, Miuta Rafila Filip, Irina Ciotlaus, Lucian Barbu Tudoran, Gheorghe Borodi, Gertrud Alexandra Paltinean, Emanoil Pripon, Claudiu Ioan Bunea

**Affiliations:** 1 Faculty of Horticulture and Rural Business Development, University of Agricultural Sciences and Veterinary Medicine, 3-5Manastur Str., 400372 Cluj-Napoca, Romania; 2 Faculty of Science, Technical University of Cluj-Napoca, 76 Victoriei Street, 430122 Baia Mare, Romania; 3 Faculty of Chemistry and Chemical Engineering, Babes-Bolyai University, 11 Arany Janos Street, 400028 Cluj-Napoca, Romania; 4 Department of Polymer Composites, Institute of Chemistry “Raluca Ripan”, Babes-Bolyai University, 30 Fantanele Street, 400294 Cluj-Napoca, Romania; 5 Department of Organic Compounds and Natural Products, Institute of Chemistry “Raluca Ripan”, Babes-Bolyai University, 30 Fantanele Street, 400294 Cluj-Napoca, Romania; 6 Faculty of Biology and Geology, Babes-Bolyai University, 44 Gheorghe Bilaşcu Street, 400015 Cluj-Napoca, Romania; 7 National Institute for Research and Development of Isotopic and Molecular Technologies, 65-103 Donath Street, 400293 Cluj-Napoca, Romania; 8 Zalau County Museum of History and Art, 9 Unirii Str., 450042 Zalau, Romania

**Keywords:** ancient sediments, archaeometry material evidence, elemental analysis

## Abstract

Methods for material investigation are powerful tools that allow specialists to elucidate important aspects regarding ancient artifacts such as the Roman amphorae deposits discovered at Potaissa Fortress in Turda, Romania. Archeological debate states that the deposit contained olive oil and wine amphorae, but no material evidence has been presented until now. The current research is focused on the most representative large amphora fragments found in the Potaissa deposit, with a significant amount of sediment on their walls, to give archeologists the material proof to elucidate their debate. Sediment was collected from each fragment and subjected to complex analysis. XRD investigation combined with cross-polarized light microscopy demonstrated mineral particles such as quartz, clay (muscovite and traces of biotite), and calcite. Quartz and calcite particles have a rounded shape and diameters in a range of 20–200 µm, and clay particles have a lamellar shape and dimensions from 1 to 20 µm, a fact confirmed by SEM microscopy. Sample 2 presented a large amount of amorphous phase followed by Samples 1 and 3, with a low amount of organic phase. FTIR investigation confirms organic phase presence owing to strong absorption bands regarding C-H, C=O, and O-H chemical bonds related to aliphatic compounds in Sample 2, and to some decayed wine residue in Samples 1 and 3. EDS elemental analysis was used for organic particle identification in the amphora sediments and to obtain a correlation with their microstructure. GC–MS investigation showed volatile compounds related to wine residue for Samples 1 and 3 and decomposed fats for Sample 2. Tartaric and malic acid were identified by HPLC in Samples 1 and 3, which are wine biomarkers. The correlation of all experimental results concludes with no doubt that Amphora 2 contained olive oil and Amphorae 1 and 3 contained wine in ancient times.

## 1. Introduction

The modern physico-chemical investigation of ancient artifacts allows advanced material characterization, which is necessary for historians to certify important aspects regarding the past [1,2,3,4]. Crystalline phases and chemical bond identification within organic molecules of ancient materials are useful to confirm or to infirm historians’ suppositions relating to various aspects involved in archeological excavations. Amphorae are the most common vessels of liquid and semi-liquid matters (e.g., wine, olive oil, grease, condiment emulsions), and were used for transport and storage during ancient times. These vessels are made from clay pottery with a certain network of pores and internal micro-fissures that allow the stored liquid to infiltrate and generate consistent sediment during their service time. Therefore, physico-chemical investigations are very valuable in ancient amphora discoveries because of their ability to track the nature of liquids that were stored inside them. There are some relevant data in the literature about amphorae discovered at Pyrgi and Castrum Novum by the Tyrrhenian Sea, where archaeometric investigation found pine resin [5]. The observed sediment resembles dark spots stacked inside the amphorae and was collected with a scalpel. Another study reports the physical and chemical investigation of wine amphorae found in a Tyrrhenian Sea shipwreck nearby Albenga, Italy [6], where pollen from Vitis Vinifera allowed the certification of their content.

The organic material within some Roman amphorae discovered in Ukraine has been DNA tested, presenting similar qualities to actual Tuscan wines in Italy, proving the ancient wine trade relationships over the Roman Empire frontier, as reported by Milanesi et al. [7]. These findings are very important because they certify that significant organic material may be found after over 2000 years. Additionally, the mineral content of the ancient amphorae presents archaeometric interest, e.g., the clay occurrence in various amphorae discovered in Alamaraz, Portugal. Different clay sediments identified by X-ray diffraction (XRD) allow the proper identification of several amphora sources in antiquity [8]. Mineral fraction investigation has proven to be a key role in understanding the archaeometric status of amphorae. Therefore XRD investigation was coupled with Fourier-transform infrared spectroscopy (FTIR) to demonstrate the chemical bonds within both the mineral and organic matter in sediment [9,10,11]. Besides clays, quartz and calcite are two of the most important mineral fractions. They could generate exogenous contamination of the amphora sediment following the long exposure of the fragments to resting soil. The exogenous contamination of sediment must be considered in the discussion.

One of the most important frontiers of the Northern Roman Empire was in Dacia Porolissensis. With the administrative center at Porolissum Fort, it played an important role in assuring frontier security and commercial exchanges with Barbarian tribes. The success of their effort depended on a goods supply chain. Military effects and domestic supplies including food and drink were delivered on the main road from Apullum—Potaissa—Napoca to Porolissum. The Potaissa Fortress was one of the most important military and commercial bases, as it was the headquarters of Military Legio V Macedonica, home to an active military commander with the rank of “Legatus Legionis”. Some recent archeological discoveries have been unearthed at the Potaissa Fortress, now situated in Turda, Romania. One of these discoveries is an amphora deposit situated in one of the commander’s residence rooms positioned on the northern side, as Barbulescu reported in 2020 [12]. Several hundred amphora fragments were found, which were classified by archeologists according to their shapes and pottery seals. The general opinion based on the historical evidence is that the amphorae were used in olive oil and wine transport on the main road in northern Dacia from Apullum to Porolissum via Napoca, but it is difficult to certify that beyond doubt. Archeologists found a few representative amphora fragments with significant ancient sediment on their inside walls. The physico-chemical investigation of these samples offers important proof to historian theories.

The aim of the present article is the investigation of the most relevant three sediments collected from the amphora fragments discovered at Potaissa, to establish the nature of their content and their interaction with the resting ground over the centuries. The null hypothesis of the present research is that no organic matter is present in the collected samples.

This is a preliminary study of the amphora sediments collected from the Potaissa Fortress and helps archeologists to continue their study of relevant evidence brought by the material investigation using modern techniques. It is the starting point for a more detailed analysis of the collected amphora sediments that will be conducted in future research that will reveal secrets hidden for more than 1800 years.

## 2. Materials and Methods

**Figure 1:**
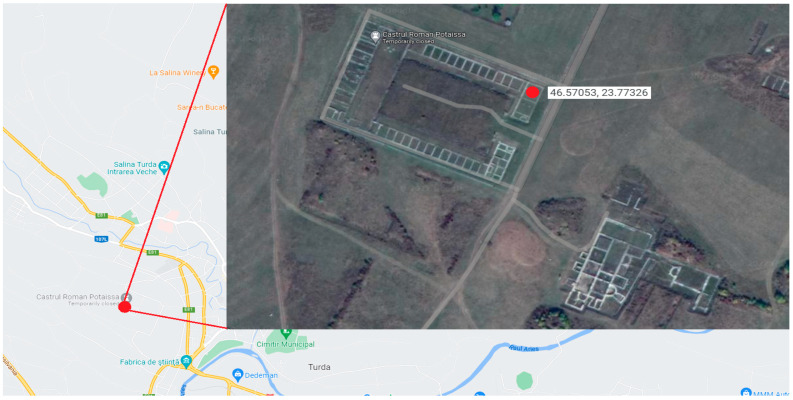
Geographical placement of the Potaissa Roman Fortress, Turda, Romania, [Google Maps view 2022] GPS coordinates: 46.57053, 23.77326.

The amphora fragments were discovered in room “P” of the *principia* building of the Potaissa Fortress, marked with a red spot in Figure 1. Some of the most representative fragments discovered present traces of ancient sediment.

**Figure 2:**
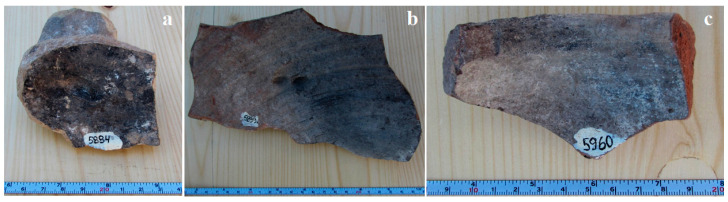
Amphora fragments: (**a**) Sample 1, (**b**) Sample 2 and (**c**) Sample3.

Powder samples were collected gently using sharp tweezers and a scalpel from three amphora fragments, presented in Figure 2, with inventory numbers: 5884 for Sample 1; 5899 for Sample 2; and 5960 for Sample 3. The collected powder samples were stored in Eppendorf vials.

Mineralogical microscopy was conducted in cross-polarized light using a Laboval 2 microscope, Carl Zeiss Jena, equipped with a Samsung 10 Mpx digital photo system. The sediment particles were spread out on glass lamella to reveal both small and large sizes. At least three different macroscopic sites on each sample were investigated.

X-ray diffraction (XRD) was conducted with a Bruker D8 Advance diffractometer using monochromatic radiation CuK_α1_ (λ = 1.54056 Å) at room temperature. The mineral phase identification was performed using Match software (Crystal Impact Company, Bonn, Germany). At least three determinations were carried out for each sample to assure a proper statistical average.

Fourier-transform infrared spectroscopy (FTIR) was carried out using an FTIR 610 spectrometer (Jasco Corporation, Tokyo, Japan), in the 4000–400 cm^−1^ wavenumber range, using the KBr pellet technique. The resolution of the spectra was 4 cm^−1^, and scans were repeated 100 times. All FTIR spectra were registered at room temperature. At least three determinations were carried out for each sample.

Sediment morphology and elemental composition were investigated with a Hitachi SU8230 scanning electron microscope (SEM) equipped with energy-dispersive spectroscopy (EDS) detector X-Max 1160 EDX (Oxford Instruments). Sample mounting on the SEM holder using carbon paste was not used in order to avoid influencing the carbon elemental counting. Therefore, sediment powder samples were immobilized onto the SEM sample holder by metallization with a thin film of gold deposited by vacuum sputtering, and the Au component within EDS spectra was subtracted. At least three different macroscopic sites on each sample were investigated.

Organic volatile compounds from the amphora sediment samples were qualitatively determined by Gas Chromatography—Mass Spectrometry (GC–MS). Samples (0.2 g) were diluted in dichloromethane (1 mL) and a volume of 1μL was injected into the GC device. An Agilent GC–MS Gas Chromatograph-7890A/5975/2008 (Agilent Technologies, Inc. Europe, Waldbronn, Germany) was used for analysis, which was performed in scan mode on a DB-5MS (30 m × 0.25 mm × 0.25 µm) capillary column (Agilent 19091S-433M), high-purity He carrier gas at a flow rate of 1 mL/min. The temperature program was as follows: initial temperature 50 °C with a ramp rate of 8°C/min up to 220 °C, then with 20 °C up to 280 °C and maintained for 10 min, injector temperature 260 °C, injection volume of 1 µL, 100:1 split ratio, MS 70 eV, mass range u.a.m. 30–400. The NIST library was used for the identification/confirmation of the component structure. Qualitative analysis was based on the area percentage of each peak of the sample compounds. The total ion current (TIC) chromatogram represents the summed intensity across the entire range of masses being detected at every point in the analysis. Sample preparation implied the methylation reaction occurred in a cooled methanolic solution of potassium hydroxide, according to the standard procedure. Samples (0.10 g) were added to a mixed solution of 3 mL of hexane and 500 μL of 2 N mol L^−1^ methanolic KOH in a test tube of 5 mL. This was stirred vigorously for 15 s, allowing the layers to separate until the top solution became clear. The upper layer containing methyl esters was decanted and dried over MgSO_4_.

High-performance liquid chromatography (HPLC) was used to search for wine biomarkers in sediment Samples 1 and 3. The analyses of organic acids were carried out on a high-performance liquid chromatograph (Jasco Corporation, Tokyo, Japan) equipped with a UV/Vis detector and injection valve with a 20 μL sample loop (Rheodyne, Thermo Fischer Scientific, Waltham, MA, USA) for the manual injection of the sample. Chrom Pass software was used to control the HPLC system and to collect and process the chromatographic data.

Separation was carried out on a Carbo Sep Coregel 87H3 polymeric column (300 × 7.8 mm), at 35 °C column temperature. The mobile phase was the 0.005 M sulfuric acid solution. The flow rate was 1 mL/min and UV detection was 214 nm. The organic acid standards (tartaric, citric, malic, succinic, and oxalic) and sulfuric acid were purchased from Merck (Darmstadt, Germany). Ultrapure water (<18.3 MΩ·cm) was prepared with Milli-Q plus using the Symplicity^®^ UV water purification system (Millipore SAS, Molsheim, France). All reagents were of analytical grade.

Sample preparation: the extraction of organic acids (tartaric and malic) from the studied samples was carried out in Millipore water. About 15 mg of the sample and 0.5 mL of ultrapure water were sonicated with an ultrasonic bath (Bandelin Sonorex, Berlin, Germany) at power 100% and 80 KHz ultrasonic frequency, at room temperature for 20 min, and then centrifuged (Eppendorf 5804 R centrifuge, Hamburg, Germany) at 4500 rpm for 10 min. Finally, the supernatant was passed through a 0.45 µm nylon syringe filter (Teknokroma) and injected into the HPLC system. All samples were analyzed in triplicate. The results were expressed in mg organic acid/100 g sample.

## 3. Results and Discussion

### 3.1. Optical Mineralogical Microscopy

**Figure 3:**
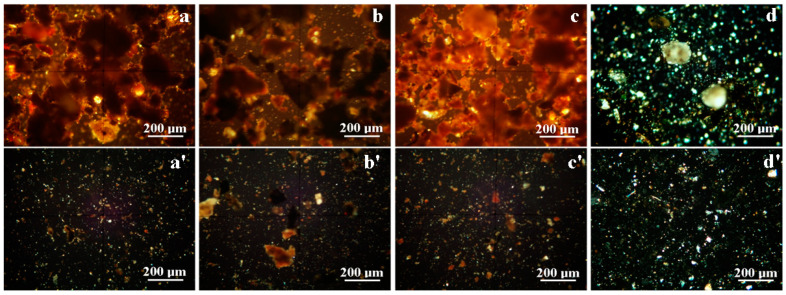
Optical mineralogical microscopy with cross-polarized light for large particlefraction: (**a**) Sample1, (**b**) Sample 2, (**c**) Sample 3 and (**d**) resting soil; and for small particle fraction: (**a′**) Sample1, (**b′**) Sample 2, (**c′**) Sample 3 and (**d′**) resting soil.

Mineralogical microscopy images obtained for Sample 1 are presented in Figure 3a for the large particles and Figure 3a′ for the smaller fractions. It contains a grainy mixture with a significant amount of bigger clusters with boulder shapes and sizes ranging from 75 to 300 μm (Figure 3a) formed due to the coalescence of clay particles such as muscovite. These are surrounded by small particles in the range of 25 to 75 μm, belonging to calcite (yellow–brown), quartz (yellow–green–gray) and some darker particles with a diameter below 5 μm corresponding to the amorphous fractions. These are more visible among fine particles in Figure 3a′.

Sample 2 presents a significant number of bigger particles (Figure 3b), with boulder shapes and sizes of about 75–220 µm, many of them being quartz and some calcite with sizes of about 20–75 μm. A significant amount of these particles is made of fine fraction clusters such as traces of muscovite bonded by organic material featuring darkened aspects in cross-polarized light. Figure 3b′ allows better observation of the rich organic particles along with fine mineral fractions with irregular shapes and sizes between 20 and 50 μm.

The cross-polarized light aspect of Sample 3, Figure 3c, reveals a higher crystallinity level among amphora sediment samples. The observed particles are smaller: quartz 10–50 μm, calcite 20–50 μm, and muscovite presenting fine fractions below 10 μm (these are more visible among fine fractions in Figure 3c′). Some organic particles also occur, with diameters between 1 and 60 μm.

Amphorae fragments were recovered from an average depth of −0.70 m, and covered with a highly mineral resting soil with a sandy texture. It is very different from the upper fertile soil, which contains plant roots and humus compounds. Therefore, the resting soil (Figure 3d) demonstrated some bigger particles of quartz and calcite particles of about 90–120 μm. They are surrounded by a mixture of small particles of quartz and calcite (10–30 μm). The finest fractions observed in Figure 3d’ reveal some quartz and calcite particles with sizes between 1 and 10 μm surrounded by a lot of muscovite particles of 10 μm and below. The mineral particles found in the resting soil bear a great resemblance to the ones observed in the sediment samples. The dominant mineral in the resting soil is quartz, but it is difficult to establish its influence on the amphora sediment sample. Therefore, XRD analysis is required to fulfill the purpose of determining the exact amount of each of the crystalline phases.

### 3.2. X-Ray Diffraction (XRD)

**Figure 4:**
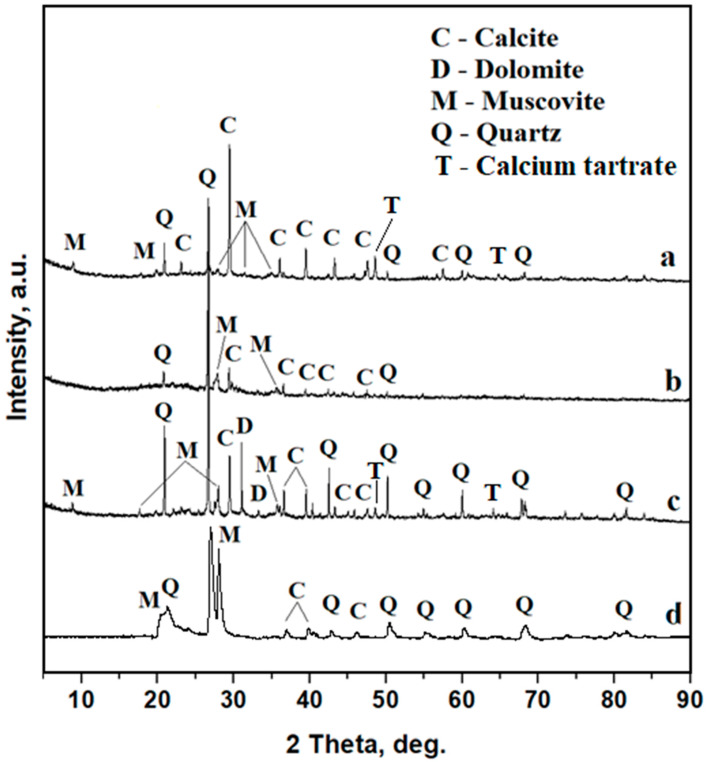
XRD patterns for amphora sediments: (**a**) Sample1, (**b**) Sample2, (**c**) Sample 3 and (**d**) resting soil.

The XRD spectra for Samples 1 and 3 feature well-developed peaks with a narrow shape corresponding to a high degree of crystallinity (Figure 4a, c). Quartz and calcite peaks are very intense and muscovite peaks show less intense peaks partly due to the small size of the diffracting particles. Some small but relevant peaks of calcium tartrate were found. This compound is related not to the mineral composition of Samples 1 and 3 but to the organic matter. Data in the literature show that tartaric acid from wine may interact with the amphora wall, causing the precipitation of small calcium tartrate crystals, known as wine biomarkers [13,14,15].

Sample 2 presents an XRD pattern with well-developed peaks for quartz and calcite and a significant influence from the amorphous material, Figure 4b.

The percentage of each phase in the samples was evaluated with the RIR (reference intensity ratio) method. This method assumes that if there are two phases in the sample, then we have Equation(1):

(1) $$\frac{Ia}{Ib}=\left(\frac{xa}{xb}\right)\cdot \left(\frac{fa}{fb}\right).$$

where: *Ia* is the intensity for the most intense diffraction peak from phase a, and *Ib* is the intensity for the most intense diffraction peak of phase b, *xa* and *xb* are the mass percentage concentrations for the two phases and *fa*, *fb* are the Corundum factors for the a and b phases.

If we also have a third phase, marked with c, then we can also write a relationship, for example, between phases a and c:

(2) $$\frac{Ia}{Ic}=\left(\frac{xa}{xc}\right)\cdot \left(\frac{fa}{fc}\right).$$

Taking into account that

(3) xa+xb+xc=100%,

then the mass concentrations of the three phases in the samples can be determined. The results of the phase analysis performed by this method are presented in Table 1 (resting soil was numbered as Sample 4 in Table 1). The method was adapted for the determination of calcium tartrate traces with in Samples 1 and 3.

The degree of crystallinity is determined as the ratio between the sum of the areas of the diffraction peaks and the sum of the areas of the diffraction peaks plus the area of the diffraction halos due to amorphous phases. We obtained 59% for Sample 1, 42% for Sample 2, and 71% for Sample 3.

Bulk calcite occurs in marble with a polycrystalline structure based on microscopic crystallites well pressed together during geological conditions [16,17]. However, calcite and micro-sized quartz particles are reported in the eroded sedimentary soils of Vitis Vinifera culture areas by Nagy et al. [18] and are a common presence in the muscovite and kaolinite clay soils as observed by Negreanu et al. [19].

The data in the literature demonstrates the constant mineral phase composition of soil in the specific area of interest. In this respect, the ground that covers the amphora fragments in room “P” has a uniform mineral composition, as observed in Figure 4d. The XRD pattern of the resting soil shows that quartz is the dominant mineral followed by muscovite and calcite. The amphora sediment mineral phase composition is very different for each of the investigated samples. It means that the resting soil influences the mineral composition of the amphora sediments only partly, and the other mineral sources may be some minor excoriations of the amphora wall during sampling and perhaps traces of soil particles from the ancient sources that were incorporated into the sediment from the original amphora content. The significant presence of dolomite in Sample 3 might be related to the amphora presence in other places in ancient times [6,20]. Some recent data shows that the Ca/Mg ratio in dolomite may be affected by regional characteristics, which may include traces of Na, Fe, Mn, and Sr due to local marine sedimentary conditions [21]. This fact will be very useful in the elemental characterization of the samples. The mineral fraction observation reveals that all three sediments contain only one carbonaceous mineral, namely calcite, and its relative blend with magnesium as dolomite was found in Sample 3. This fact is very important for the carbon balance during the EDS investigation of organic compounds.

Another important mineral finding is α quartz occurrence in Sample 2. This is a hexagonal allotropic form of quartz resistant to high temperatures. Data in the literature show its occurrence in clay pottery fired at lower temperatures that prevent the quartz’s transformation into tridymite [22,23]. It proves that some of the mineral particles in the collected sediments belong to the amphora walls.

### 3.3. Fourier-Transformed Infrared Spectroscopy (FTIR)

**Figure 5:**
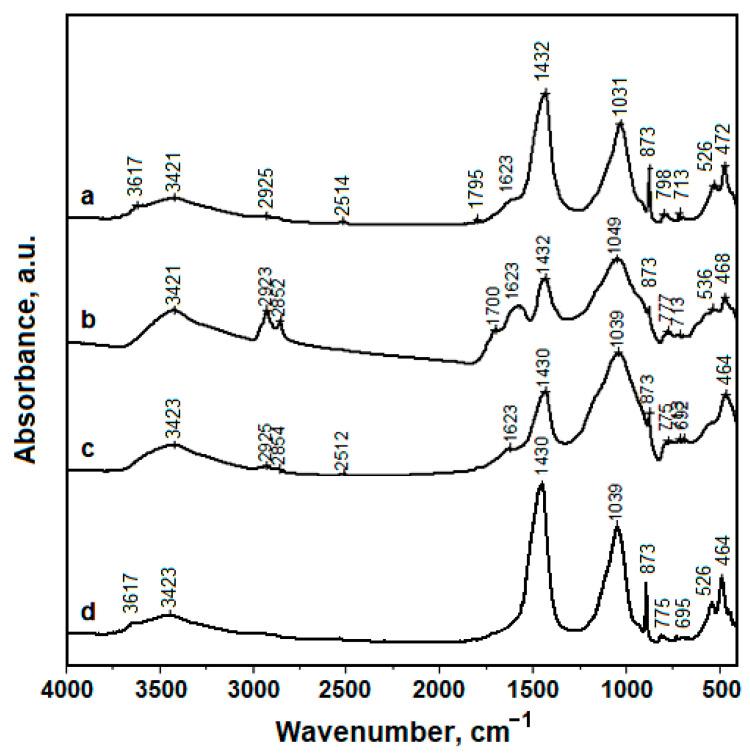
FTIR spectra results in amphora sediments: (**a**) Sample1, (**b**) Sample2, (**c**) Sample 3 and (**d**) resting soil.

Chemical bonds within the sediment samples were investigated with FTIR spectroscopy and the resulting spectra are presented in Figure 5. The chemical bonds within mineral fractions are situated on lower wave numbers such as silicates and phyllosilicates [10,24]. Muscovite is the phyllosilicate found within amphora sediment samples. The richest one is Sample 1, followed by Sample 3, and Sample 2 has only traces, as observed by the XRD results (Table 1). Its formula ([K_2.62_Na_0.13_Ca_0.01_](Al_3.08_Fe^III^_0.10_Mg_0.10_Fe^II^_0.27_)(Si_6.65_Al_1.35_)O_20_(OH)_4_) reveals the complexity of the chemical bonds involved. Therefore, the most characteristic FTIR features of muscovite in the recorded spectra are the broad band around 1030 cm^−1^ related to in-plane Si-O stretching and the doublet at 530 and 470 cm^−1^ related to the Si-O-Si bending mode, which is in good agreement with the literature [25,26]. The other functional groups with stretching mode are hydroxyl bonds at 3617 cm^−1^ (strong band) and 3421–3423 cm^−1^ (shoulder bands), Al–O–H stretch 1575–1795 cm^−1^, Si-O-Si bonds at 692–796 cm^−1^, the in-plane vibrations associated with Si–O–Al bonds at 775–798 cm^−1^, and the vibrations of the apical Al–O bond from the tetrahedron at 873 cm^−1^.

Absorption bands observed at 775–798 cm^−1^ belong to the symmetric deformation of CO_3_^2−^; 873 cm^−1^ to asymmetric CO_3_^2−^ bending; 1430–1432 cm^−1^ corresponds to asymmetric CO_3_^2-^ stretching; and all are related to the calcite occurrence in mineral fractions [10,27,28].

The H-O-H bonds related to the adsorbed water also occur at 3423–3421 cm^−1^, most likely due to water filtering into the clay particles [29,30]. The resting soil where the amphora fragments were found was subjected to repeated exposure to the water from rainfall infiltrating the soil, and therefore it is normal for water to have absorbed mainly into small fractions of the muscovite particles [29,30].

The significant absorption bands at 1623 cm^−1^ demonstrated for Sample 2 (and less intensely for Samples 1 and 3) correspond to the asymmetric stretching of C=O related to the presence of carboxylic acid salts as reported in the literature [10,31,32].

The absorption bands at 2923–2925 cm^−1^ belong to CH_2_ asymmetric stretching, and 2852–2854 cm^−1^ belongs to CH_2_ symmetric stretching in strong connection with aliphatic compounds [9,11]. The one found at 1700–1795 cm^−1^ belongs to C=O stretching [10,31,32]. This is a strong organic characteristic, which may occur in various compounds. Some interesting data in the literature relates these absorption bands to vegetable resins [20]. This fact also agrees with the fact that pine resin was used to seal wine amphorae in order to assure waterproof transport [5,7].

FTIR investigation of the sediments collected from amphora fragments completes the XRD and mineralogical microscopy analysis, and allows a more precise view of the content kept in the amphorae. It seems that Amphorae 1 and 3 were used for wine and Amphora 2 was used for olive oil. Validation of these findings may be obtained using a complex high-resolution microscopic analysis coupled with an elemental investigation of the sediment particles.

### 3.4. Scanning Electron Microscopy (SEM) and Elemental Analysis(EDS)

**Figure 6:**
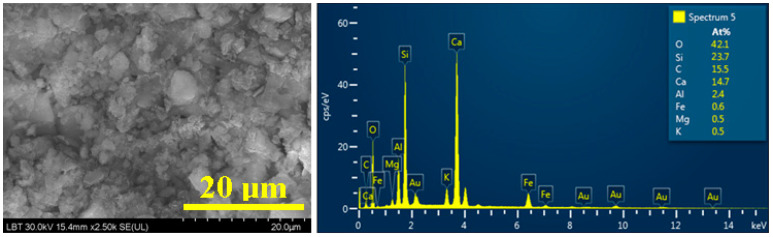
SEM image for the resting soil and the corresponding EDS spectrum.

High-resolution SEM microscopy coupled with an energy-dispersive spectroscopy module (EDS) is a powerful tool able to investigate complex structures containing mineral and organic phases [33,34], and it is very specialized in detecting trace elements in organic matter [35,36]. Therefore, SEM and EDS analysis was structured in three steps: the first step is focused on the investigation of resting soil (Figure 6), followed by a detailed view of the mineral particles, and the chasing of the organic particles.

Resting soil demonstrates a particle conglomerate formed mainly of quartz particles with a boulder shape and sizes varying from 3 to 8 μm surrounded by the finest muscovite particles with dimensions between 1and5 μm (Figure 6), and some calcite particles as small rounded boulders with dimensions about 2.5–3 μm. This fact is in good agreement with the optical microscopy and XRD results. The EDS spectrum in Figure 6 reveals Si to be the dominant element due to the high amount of quartz and muscovite. Aluminum and potassium are related to the muscovite particles as well as Fe and Mg, which often occur as inclusions in muscovite. Calcium and carbon amounts are very close to the calcite stoichiometry, which confirms the XRD results.

**Figure 7:**
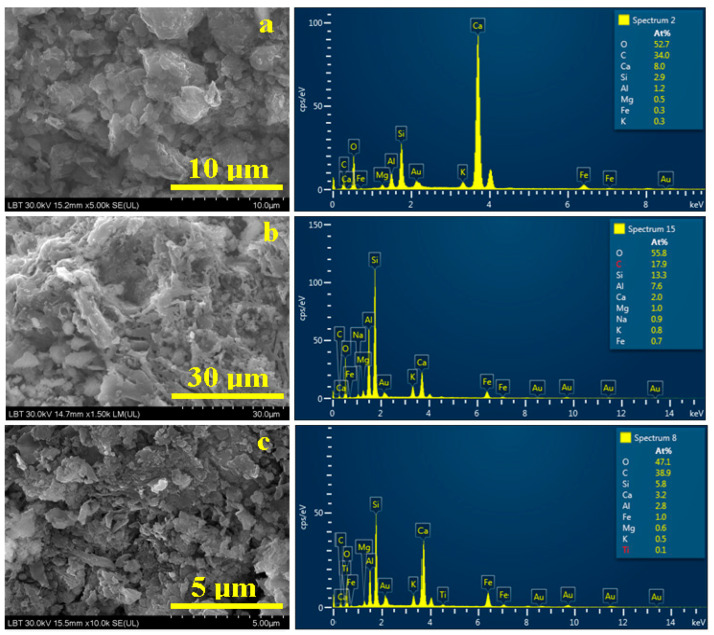
SEM images and EDS spectra obtained for the mineral fractions within the amphora sediments: (**a**) Sample1, (**b**) Sample 2 and (**c**) Sample 3.

SEM imagery of the mineral phases from Sample 1 (Figure 7a) demonstrates a particle conglomerate. It contains calcite (the most relevant calcite particle is about 6 µm and is situated in the center of the observation field, and a similar one is situated just above it) and quartz particles that are smaller (about 2.5–4 µm with a boulder shape).

Muscovite particles are the finest mineral fractions with dimensions of less than 2.5 µm and surround by bigger particles. The elemental composition is presented in Table 2. 

The dominant elements are O (52.7%), C (34%), and Ca (8%), which are related mainly to calcite. This fact is in good agreement with XRD patterns. Considering the calcite chemical formula CaCO_3_ results in 8% Ca bonds, only 8% of the carbon content and the remaining 26% belongs to the organic phase, which is not visible among mineral particles. Therefore, it is supposed that it is very small and well dispersed, and not visible in Figure 7a. The silicon, aluminum, magnesium, iron, and potassium amounts measured in Sample 1 belong to quartz and muscovite. Magnesium and iron are not common in pure muscovite, but their presence is explained by small traces of biotite (also known as black mica) [37].

Sample 2 proves to be very complex, containing large organic material crusts strongly adherent to the amphora wall. They were broken during the collecting procedures and therefore present two sides: one that was in contact with resting soil (mineral particles are trapped on its surface) and the other, which was oriented on the amphora wall. The aspect of the exterior side is visualized by the SEM image in Figure 7b. There appear to be minerals from the resting soil such as quartz particles (with diameters of about 5 µm, being situated on the lower left side of the observation field in Figure 7b) and fine muscovite particles interlocked with calcite small fractions ranging from 1 to 2.5 µm in diameter. The elemental composition reveals only 17.9% C, which is divided between calcite content of 2% and organic matter of 15.9%. It seems that the organic background has a great influence on the embedded mineral particles, a fact that is in good agreement with XRD observation. The amounts of Si, Al, K, Mg, and Fe are related to the presence of clay particles—mainly muscovite but also some traces of biotite.

The sediment within Sample 3 (Figure 7c) also presents a particulate conglomerate. These particles are the finest fractions that were observed during mineralogical microscopy. We remark that quartz with boulder shapes and sizes ranging between 1.5 and 2 µm are mixed up with calcite and dolomite particles of about 1.5 µm in diameter. Muscovite platelets represent the finest mineral fractions, with sizes of about 1 µm. The morphologic aspects are in good agreement with the elemental analysis: the calcium and magnesium amounts in Table 2 belong to the calcite and dolomite, and the silicon amounts are divided between quartz and muscovite (which also contain Al, K, and traces of Fe related to the weak presence of biotite).

**Figure 8:**
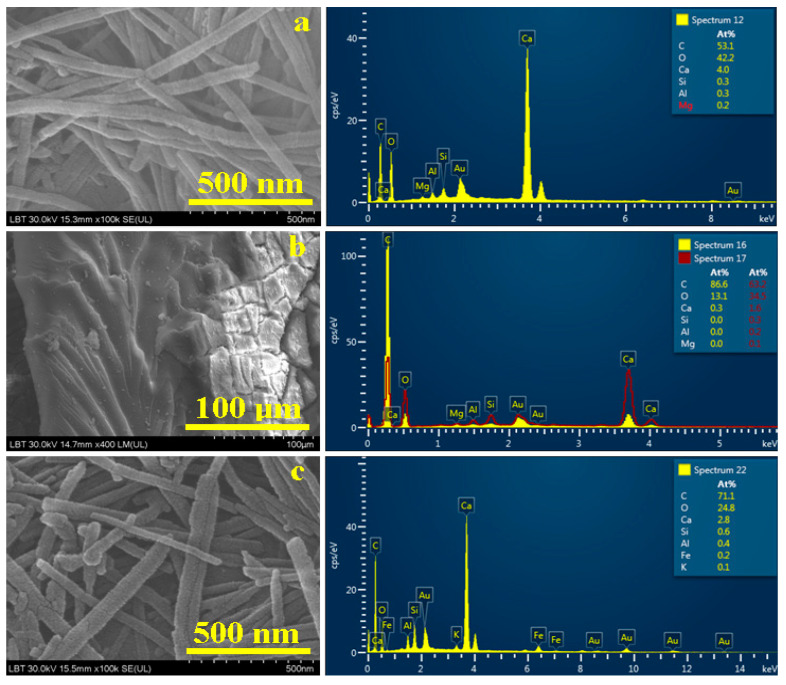
SEM images and EDS spectra obtained for the organic fractions within the amphora sediments: (**a**) Sample1, (**b**) Sample 2 and (**c**) Sample 3.

The organic particles within Sample 1 were successfully observed only at higher magnification (Figure 8a). There is a cluster of filiform nanoparticles of about 500 to 700 nm in length and 50 nm in diameter.

The EDS spectrum reveals they contain 53.1% C, 42.2% O, and 4% Ca, which prove their organic character (Table 3) and are related to the calcium tartrate crystals revealed by XRD. Their precipitation onto the mineral particle surface is in good agreement with the mechanism presented in [13]. The traces of 0.3% Si, and 0.2% Mg belong to the mineral influence of the surrounding areas, which consumes only 4% of the C amount in the chemical bonding with Ca to form the calcite structure. The filiform shapes of the observed nanoparticles resemble the fibrous illite (a mineral similar to muscovite) [38,39] but illite particles are microstructured, not nanostructured, and contain silicon and aluminum, not carbon and oxygen. Therefore, strong evidence of wine biomarker nanoparticles is found in Sample 1.

Figure 8b reveals the organic particles from Sediment 2 in a particular position due to the stage of proper position. On the right side of the observation field (Figure 8b), we observe the surface of the organic sediment crust with mineral particles embedded, and in the center and left side we observe the organic material microstructure. This resembles bituminous matter. This fact is in good agreement with the elemental composition of 86.6% C and 13.1% O, with only 0.3% Ca (Table 3). These aspects relate to the aliphatic CH_2_ asymmetric stretching and the CH_2_ symmetric stretching observed in the FTIR spectrum of Sample 2, which leads to the conclusion that amphora 5960 (Sample 2) contained olive oil in ancient times. It is a certain proof that confirms archeologists’ beliefs regarding the *principia* room “P”, which stored together wine and olive oil amphorae.

SEM imagery obtained for the organic particles found in Sediment 3 is presented in Figure 8c. The shape and size are very similar to those found in Sediment 1, with particle length ranging from 600 to 800 nm and diameter between 40 and 80 nm. The elemental composition is given in Table 3, the dominant element being carbon, with an atomic percentage of 71.1%, followed by 24.8% oxygen and only 2.8% C, a situation that’s in good agreement with XRD observations of calcium tartrate crystals.

Tartaric acid within ancient wine interacts with the mineral particles within the amphora wall, facilitating the precipitation of small calcium tartrate nanoparticles. This is strong evidence of wine residues observed in Sample 3, a fact in good agreement with the data in the literature [13,14,15]. A small influence from the finest mineral particles, such as quartz and muscovite traces situated in close proximity to organic particles, explains small amounts of Si, Al, K, and Fe.

The archeological investigation of the amphora fragments found in room “P” from the *principia* building of the Potaissa Fortress reveals with no doubt that the liquids stored in those recipients were wine and olive oil. Therefore, the sediment found in Sample 2 belongs with no doubt to olive oil remains; its morphology is totally different from the organic particles found in Samples 1 and 3. Therefore, there is no doubt that Sediments 1 and 3 could not be olive oil remains, and by elimination, they should belong to ancient wine remains.

### 3.5. Gas Chromatography-Mass Spectrometry (GC–MS)

The combined results obtained by FTIR and SEM-EDS analysis give important information regarding the organic matter within amphora sediment samples, but are not enough to confirm the presence of wine in Sediments 1 and 3. The scarcity of organic matter within these samples makes the problem more difficult. Therefore, a more sensitive method is required—a method that implies solvent extraction and sensitive measuring. Such a method is the GC–MS, which can detect small traces of volatile compounds resulting from organic fractions of the amphora sediments. Sample storage in plastic Eppendorf vials caused important contamination with phthalates, affecting the mass spectrometry quantitative determination. Therefore, phthalate peaks were not considered, and the obtained MS values for relevant peaks give only a qualitative description of the samples.

**Figure 9:**
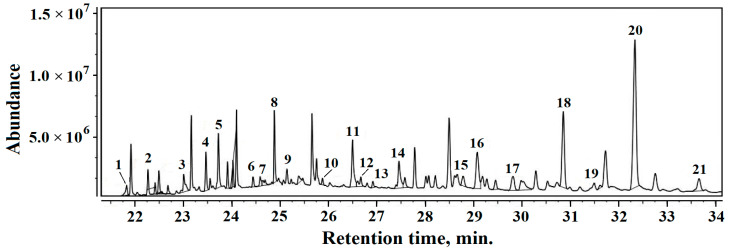
TIC chromatogram results for organic fraction within Sample 1.

The volatile compounds identified in Sample 1 are as follows, Figure 9, (relative percentages are given in brackets): 1. Octanoicacid, hexyl ester (0.74%), 2. Isopropyl myristate (1.19%), 3. 1-Hexadecanol (1.27%), 4. Homosalate (1.84%), 5. Octanoic acid, dodecyl ester (3.26%), 6. Isopropyl palmitate (1.05%), 7. 2-Undecenal (0.63%), 8. Isopropyl stearate (0.67%), 9. 1-Dodecanol, 2-octyl (0.45%), 10. Tridecane (0.55%), 11. Tetradecane (3.47%), 12. Hexadecane (0.77%), 13. Hexanedioic acid, dioctyl ester (0.30%), 14. Heptadecane (0.79%, 15. Glycerol tricaprylate (1.20%), 16.Eicosane, 10-methyl (3.68%), 17. Eicosane (0.71%), 18. Octocrylene (6.67%), 19. Heneicosane (0.34%), 20. Squalene (16.94%), 21. Pentadecane, 8-hexyl (1.49).

**Figure 10:**
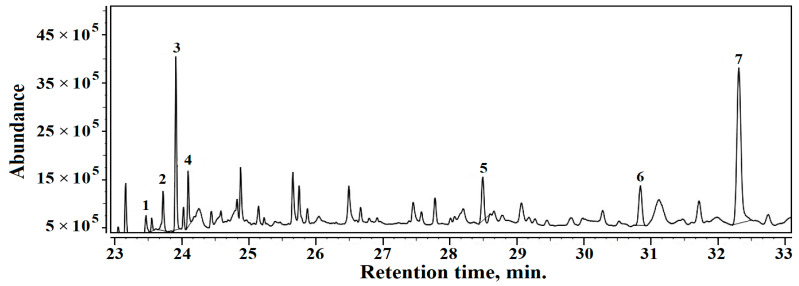
TIC chromatogram results for organic fraction within Sample 2.

GC–MS chromatogram results for Sample 2 are presented in Figure 10. The volatile compounds identified in Sample 2 are as follows (the relative percentages are given in brackets): 1. Homosalate (1.85%), 2. Cyclodecane (4.34%), 3. Dibutyl phthalate (10.57%), 4. Isopropyl palmitate (1.24%), 5. Bis (2-ethylhexyl phtalate) (4.96%), 6. Octocrylene (5.51%), 7. Squalene (26.96%).

**Figure 11:**
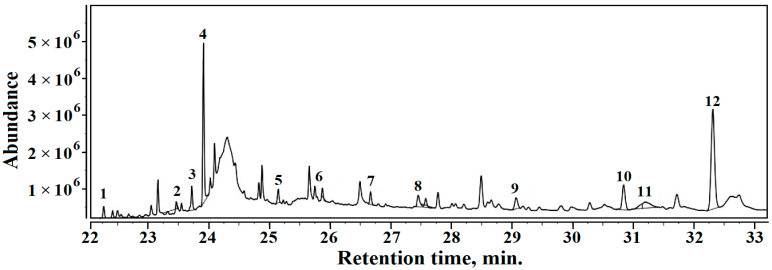
TIC chromatogram resulted for organic fraction within Sample 3.

The GC–MS chromatogram results for Sample 3 are presented in Figure 11. The volatile compounds identified in Sample 3 are as follows (the relative percentage is given in brackets):1. Isopropyl myristate (0.64%), 2. Homosalate (0.81%), 3. Butyl decyl ether (1.31%), 4. Isopropyl palmitate (2.60%), 5. Tridecane (0.94%), 6. Tetradecane (1.13%), 7. Hexadecane (0.99%), 8. Eicosane (0.81%), 9. Bis (2-ethylhexyl phtalate) 2.80%).10. Heneicosane (1.84%), 11. Octocrylene (2.71%), 12. Squalene (13.10%).

The dominant organic volatile compound in all three samples is squalene, which confirms the sediment origins in the amphora’s ancient content. Unfortunately, squalene is reported in the literature to have a strong connection with not only wine residue [40,41] but also olive oil [42,43]. Thus, several volatile compounds such alcohol residue, such as 1-Hexadecanol and 1-Dodecanol, 2-octyl and several alkanes such astridecane, teradecane and heptadecane were found in Samples 1 and 3 which do not appear in Sample 2. These are also related to the distillation process and wine residue, and data in the literature relate their presence to some sorts of wine [44,45,46,47].

The wine residue related to volatile compounds found in Samples 1 and 3 is accompanied by moderate amounts of oil derivatives, which are characteristic of Sample 2. It presents a combination of organic derivatives such as homosalate, isopropyl palmitate, and octocrylene, which are long related to olive oil, decomposing in the presence of alkaline minerals such as calcite and muscovite. No traces of wine-related alkanes were found in Sample 2. Chassouant shows that, in Roman times, amphora fragments were deposited all together in special places [41]. Contact between different fragments might have facilitated oil spillage from several fragments onto wine amphora fragments, which would generate local contamination. Wine residue would rapidly become dry just after the amphora fragment dump disposal (e.g., Fragments 1 and 3), while the olive oil on the other amphora fragments (e.g., Fragment 2) would remain liquid for long enough to facilitate a local spillage. This scenario regarding amphora fragment dumping is sustained by the data in the literature [41,48,49].

### 3.6. High-Performance Liquid Chromatography(HPLC)

**Figure 12:**
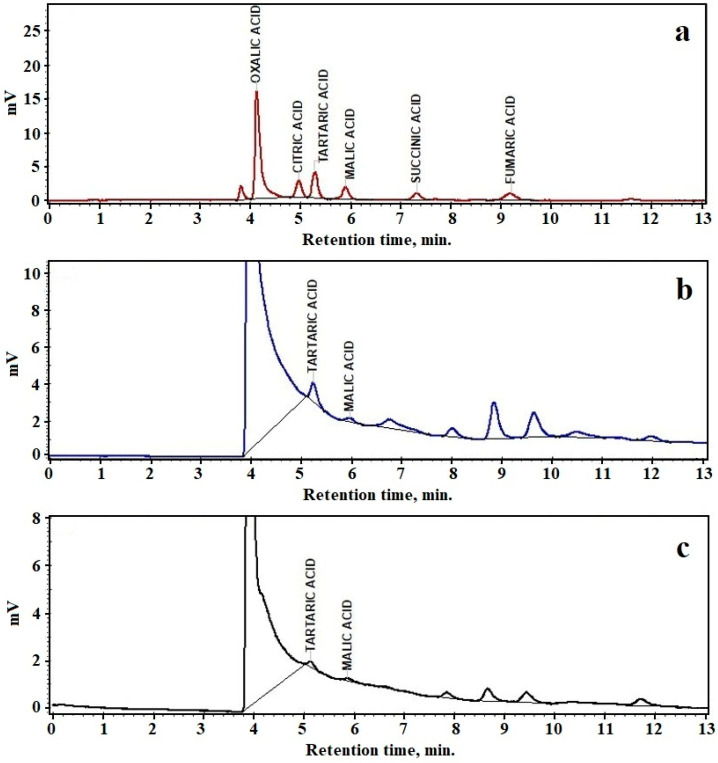
HPLC chromatograms: (**a**) standard reference, (**b**) Sample 1 and (**c**) Sample 3.

HPLC chromatograms obtained for amphora sediment Samples 1 and 3 are presented in Figure 12. The organic acid quantification was conducted by the method in [50].

The results show that amphora Sediment 1 contains 32.81 mg of tartaric acid followed by 10.59 mg of malic acid in 100 g of sample. Sediment 3 contains 11.77 mg of tartaric acid and 4.86 mg of malic acid in 100 g of sample. A significant amount of tartaric acid identified in Samples 1 and 3 is in good agreement with the XRD and SEM-EDX observations.

The long-time storage of wine biomarkers via precipitation with calcium into the amphora wall’s pores is an effective way to preserve viable information through the centuries. A high level of water solubility allows HPLC to detect the stored tartaric and malic acids, proving without any doubt that Amphorae 1 and 3 were used for wine storage in ancient times. Malic acid is the second biomarker related to wine storage [13,14], related more to red wines than white ones [51,52,53]. This observation is in good agreement with the historical information about red wine used by Roman legions.

## 4. Conclusions

The physico-chemical investigation of the sediment samples collected from ancient amphora fragments discovered at the Potaissa Fortress confirms with no doubt that the organic matter found in Sample 2 belongs to ancient olive oil residues, a fact sustained by FTIR and GC–MS analysis. The organic matter particles found in Samples 1 and 3 are nanostructured with a length of about 500–800 nm and a diameter of about 40–80 nm with an elemental composition formed of 68.3% C, 24.8% O, and a low amount of calcium, which is in good agreement with the calcium tartrate crystallites identified by XRD accompanied by trace elements from mineral fractions. The presence of calcium tartrate revealed by XRD is sustained by HPLC chromatography, which demonstrates the relevant peaks of tartaric and malic acids in both Samples 1 and 3, which are wine biomarkers. Additionally, GC–MS results found a significant amount of alkanes related to wine residues allowing the conclusion that, from Samples 1 and 3, amphorae stored wine in ancient times. The mineral phase distribution in the sediment samples shows that their occurrence is due to a particular combination of the following sources: mineral particles from the resting soil; mineral particles abraded from the amphora wall during sediment sampling; and traces of ancient soil belonging to the geographic area where the ancient wine was produced. Therefore, each of the investigated sediments has its own mineral characteristic.

## Figures and Tables

**Table 1:** Mineral phases found in the amphora sediments (average and standard deviation).

Sample	β Quartz Wt.%	α Quartz Wt.%	Calcite Wt.%	Muscovite Wt.%	Dolomite Wt.%	Ca Tartrate Wt.%
1	31.2 ± 2.5	-	46.3 ± 3.7	20.2 ± 2.3	traces	2.3 ± 0.3
2	80.0 ± 5.5	8.8 ± 0.6	11.2± 0.9	traces	traces	-
3	63.8 ± 4.7	-	11.8 ± 1.0	4.6 ± 0.3	18.2 ± 1.2	1.7 ± 0.2
4	42.9 ± 3.2	-	25.5 ± 1.8	31.2 ± 2.2	-	-

**Table 2:** Elemental compositions of the mineral fractions within amphora sediments.

Samples	Elemental Composition, at.%								
	O	C	Ca	Si	Al	K	Mg	Fe	Ti
Sediment 1	52.7	34.0	8.0	2.9	1.2	0.3	0.5	0.3	-
Sediment 2	55.8	17.9	2.0	13.3	7.6	0.8	1.0	0.7	-
Sediment 3	47.1	38.9	3.2	5.8	2.8	0.5	0.6	1.0	0.1

**Table 3:** Elemental compositions of the organic fractions within amphora sediments.

Samples	Elemental Composition, at.%								
	O	C	Ca	Si	Al	K	Mg	Fe	Ti
Sediment 1	42.2	53.1	4.0	0.3	0.3	-	0.2	-	-
Sediment 2	13.1	86.6	0.3	-	-	-	-	-	-
Sediment 3	24.8	71.1	2.8	0.6	0.4	0.1	-	0.2	-

## Data Availability

Not applicable.
